# Can't surf, won't surf: The digital divide in mental health

**DOI:** 10.3109/09638237.2012.689437

**Published:** 2012-06-19

**Authors:** Liam Ennis, Diana Rose, Mike Denis, Ninjeri Pandit, Til Wykes

**Affiliations:** 1 Health Services and Population Research Department, Institute of Psychiatry, London, UK; 2 South London andMaudsley NHS Foundation Trust, London, UK; 3 Psychology Department, Institute of Psychiatry, London, UK

**Keywords:** service delivery issues, e-health

## Abstract

*Background:* New health information technology (HIT) increasingly plays a role in health care as technology becomes cheaper and more widespread. However, there is a danger that those who do not use or have access to technology will not benefit from HIT innovations, thus creating a “digital divide”.

*Aims:* To assess the extent to which mental health service users have access to, skills in using and appetite for various technologies.

*Methods:* A cross-sectional survey was used to assess technology use and access patterns of 121 people from community mental health services. Data were analysed using logistic regression.

*Results:* Technology use and access were very similar to that of the general population with older individuals reporting less familiarity, access and confidence across a range of technologies. Black, minority and ethnic (BME) groups were more likely to access computers outside of their own homes than white individuals. Older participants experiencing psychosis indicated a desire to increase their computer use.

*Conclusions:* The findings reported here contrast with recent evidence suggesting that those who do not engage with technology are “self-excluders”. Furthermore, BME groups may need extra support regarding provision of technology in order to engage with HIT.

## Introduction

Investment in health information technology (HIT) in both the USA and the UK is high because it is hoped that innovations such as computerised therapy, telehealth, electronic personal health records and Internet-based service user forums will reap substantial benefits in terms of efficiency, patient safety and quality of care, as well as financial savings (Cavanagh & Shapiro, 2004; Chaudhry et al., 2006; Ennis et al., 2011). It is clear, however, that these benefits can only be achieved to the extent that service users have the ability to engage with such systems. Whilst consumer goods indices show that technology ownership tends to be high in both the USA and UK (Office for National Statistics, 2011; Smith, 2010), there is certainly a “digital divide” between different demographic groups. It has been reported in the USA that technology use and access tends to be lower amongst less well-educated and older individuals (Fox & Vitak, 2008), those with medical conditions and minority populations (Wang et al., 2011). The most recent US census statistics show that Internet use is inversely related to the level of education, and that there is lower Internet use amongst those who are unemployed (US Census Bureau, 2010).

Mental health service users may particularly benefit from HIT as they are typically involved with services for substantial periods of time, during which they could see a number of professionals working in different spheres. HIT could provide continuity when moving between services – something which has often been a problem in mental health (Burns et al., 2009). However, little is known about technology use patterns amongst the mental health population in the UK.

Research conducted by the UK Office of Communications (OFCOM, 2009) suggests that those who do not use the Internet fall into two broad categories: those who are self-excluded and those who are resource excluded. The self-excluded group are the larger of the two categories, making up 42% of all individuals who do not have the Internet at home. This group are profiled as generally being older, with 61% having never used a computer. These individuals frequently struggle to think of the benefits of having the Internet at home. Most share a sense of indifference and this, rather than lack of skills or finances, fuels non-adoption (Coleman et al., 2010). It is not clear whether people with mental health problems might be excluded from HIT because of skills and/or access, whether finances are barriers to technology use, or whether people simply do not wish to engage with technology. The present study aims to assess these issues amongst a sample of those experiencing severe mental illness in the UK and to establish whether there is an appetite to increase the use of technology. This will help provide guidance on resource and training considerations when introducing new HIT in mental health.

## Method

### Design

A cross-sectional design was used to conduct structured interviews. The main research question was “which demographic variables predict technology access and use amongst a sample of those experiencing mental health problems?” We were particularly interested in those who have severe mental health problems as these usually predict long term contact with services.

### Measures

(1) *Use of technology schedule:* The schedule is a semi-structured interview consisting of six questions relating to technology use based on previous literature:
  (a) Incorporated technologies
    In devising this schedule, the first task was to decide which technologies to include.
    - *Mobile phones* (any mobile phone with at most, limited web connectivity) were included as they represent a low-cost, instant form of transmitting (albeit basic) HIT with high levels of population access. Research has already shown that text messages have been successful in the management of long-term conditions such as diabetes (Cole-Lewis & Kershaw, 2010).
    - *Computers*, including laptops and netbooks, were included as they have the greatest breadth of HIT delivery, capable of transmitting a huge variety of HIT such as electronic health records, computerised therapy and psychoeducation. Whilst certain HIT such as user forums are reliant upon Internet access – rather than computers *per se* – it was felt that computer use was a more appropriate question for this study as many HIT (e.g. computerised cognitive behavioural therapy packages) do not require the Internet. Furthermore, as the cost of the Internet falls, “computer access” becomes increasingly synonymous with “Internet access”; in fact, virtually all homes with a computer are currently connected to the Internet (OFCOM, 2011).
    - Two popular forms of *smartphones* (the BlackBerry and the iPhone) were included in the schedule because smartphone ownership is rapidly on the rise. Smartphones combine the convenience of a mobile phone with the flexibility of a computer, meaning that advanced HIT can be delivered on the move.
    - *Personal digital assistants* (PDAs) were the final technology included because although they may be somewhat outdated, PDAs have shown feasibility and validity when used for time sampling of patient experiences (Kimhy et al., 2006), and as such may offer future utility in the delivery of HIT.
  (b) Dependent variables
    Training and delivery of support for HIT are dependent on a number of different considerations. Security of information is also affected by the location of use, as sensitive information may be accessed in public places. The questions asked include:
  (1) Familiarity
  (2) *Ease of access:* This was felt to be a more important question than personal ownership, as lack of ownership does not necessarily confer difficulty in access to potential HIT.
  (3) Location
  (4) Frequency of use
  (5) *Confidence:* Confidence in using the technologies was an important consideration as confidence is a significant contributor to perceived ease of use of such systems, which consequently influences technology acceptance and usage (Venkatesh, 2000).
  (6) *Desire to increase use:* The final dependent variable incorporated into the schedule was desire to increase use. This was because intrinsic motivation is another important indicator of technology use and acceptance (Venkatesh, 2000).

A service user advisory group amended the questionnaire to ensure ease of understanding. In addition to specific answers to the structured question, relevant comments were also recorded.

(2) *Sociodemographic and clinical variables* included age, time in contact with mental health services, gender and ethnicity (white vs. black, minority and ethnic (BME) groups). A fifth variable, case note diagnosis, was also devised with three categories: (i) psychosis: early intervention (EI); (ii) psychosis: normal community mental health team (CMHT); and (iii) other mental health problem. Psychosis was investigated in isolation from other mental health problems as it can be a particularly debilitating condition which often disrupts work and education.

### Participants

As those in minority communities are often “technology poor”, the sample was drawn purposively from an area with a large minority population. We also wished to include those with a serious mental illness as they have ongoing problems which are likely to result in the need for mental health input. We were interested specifically in those with a diagnosis of psychosis as they are likely to have particular difficulties; their problems not only start early in life but are also associated with higher levels of disability and so purposively sampled for this diagnosis to attain groups of 50 per care area. We also recruited others who were willing to take part in order to compare them with those who had a diagnosis of psychosis.

Recruitment was from two types of service which engage people at different stages on the care pathway (CMHTs and EI services who care for younger clients). Teams were based in urbanised communities that contain high proportions of BME individuals (32.4% and 34.1% respectively) and the final sample was representative of those accessing these types of services both locally and nationally.

All participants were sampled from outpatient services during March and April 2011. EI teams required clients to be between 18 and 35 years of age and with a recent onset of psychosis in the previous 12 months. The eligibility criteria for CMHTs are being 18–65 years old and experiencing serious mental health problems.

Participants were sampled through contact with their care coordinator. Ethics approval number: 10/H0722/79.

### Statistical analysis

#### Statistical power

We were interested in finding moderate not small effects that might need to be taken into account in the development of electronic health systems. As we were investigating the effects of both categorical variables (BME, gender and mental health grouping) and continuous variables (age, duration of contact with mental health services) logistic regression analysis was seen as the most appropriate method. A two-group *t*-test showed that with a sample size of 50 per group, an effect side of 0.655 could be detected with 90% power, *p* = 0.05, two-sided. This determined our purposive sampling in order to have at least 50 people in each chosen grouping.

Descriptive statistics were calculated in order to find overall levels of technology use and access amongst the sample and to assess the distributions.

The dependent variable was calculated as the response given to each individual item on the use of technology schedule. As most of the items consisted of only two possible response categories, binomial logistic regressions were fitted to the data set in order to establish whether any variables predicted item responses. Consequently gender, age, diagnosis and BME grouping were entered as covariates, with gender, diagnosis and BME grouping assigned as categorical variables with all variables entered simultaneously. Duration of contact with mental health services was not included as a predictor variable as it was highly related to age (Pearson's *r* = 0.70, *p* < 0.01).

Where item responses consisted of more than two categories, multinomial logistic regression was used entering all variables simultaneously and using the last category as the reference category. Where data were highly skewed to just one or two responses, categories were collapsed and binomial logistic regressions were used as with the binary variables.

All analysis was conducted using SPSS version 15.0.

## Results

One hundred and twenty-one participants experiencing mental illness were recruited to the study. Fifty-one of these were recruited from EI in psychosis units whilst the remaining 70 were recruited from standard CMHTs. Of these 70, 49 had received a diagnosis of psychotic disorder, with the remaining 21 experiencing some other form of mental health problem. Demographic information including means, standard deviations and percentages for the sample are shown in Table I.

**Table I:** Descriptive statistics of the study sample.

	Psychosis type			
		
Variable	Non-psychosis diagnosis (N=21)	EI (N=51)	Normal CMHT (N=49)	Total
Age, mean (standard deviation)	41.76 (8.40)	26.04 (5.33)	43.92 (9.24)	36.01 (11.46, range18–65)
Duration of illness, mean (standard deviation)	9.12 (11.96)	2.75 (2.35)	17.96 (9.54)	9.95 (10.51, range 0–39)
Gender, female *N* (%)	11(52.4)	13(25.5)	16(32.7)	40(33.1)
Ethnicity, *N* (%)				
BME	3(14.3)	39(76.5)	24(49.0)	66(54.5)
White	18(85.7)	12(23.5)	25(51.0)	55(45.5)

**Figure 1:**
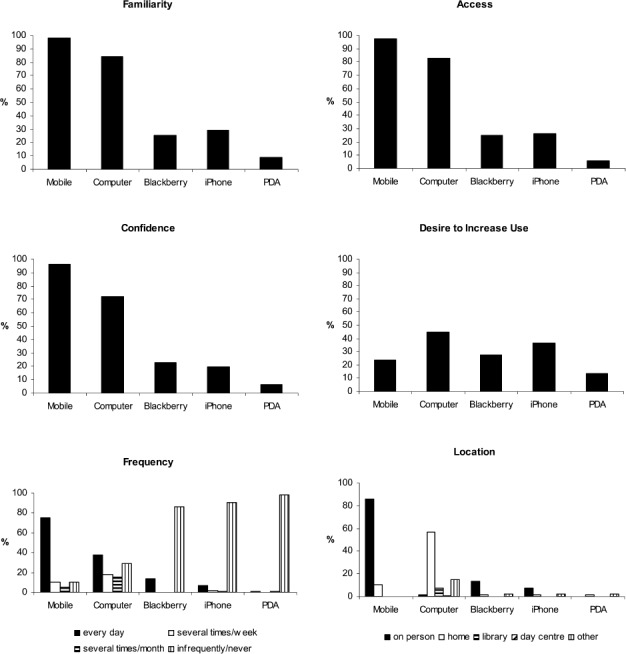
Percent of total sample who were familiar with, had access to, had confidence in using and desired to use the named devices more frequently, as well as the location and frequency of their use.

Figure 1 shows the percentages of the total sample who were familiar with, had easy access to, were confident in using and desired to use the devices more frequently, as well as the location in which these items were most commonly used and the frequency with which they were used. We found that mobile phone use was extremely high amongst our sample, with close to 100% of participants reporting familiarity, easy access and confidence. Computer use was also very high, with over 80% participants reporting familiarity and easy access and 72% reporting confidence. Around a quarter of our sample reported familiarity and easy access to smartphones (iPhones and BlackBerrys). PDA use was infrequent amongst the sample and thus the device was excluded from further analysis.

Table II shows the results of the logistic regressions. Increasing age corresponded to: less familiarity with BlackBerrys and iPhones; less access to computers, BlackBerrys and iPhones; and reduced frequency of use of mobile phones. Gender predicted mobile phone confidence, with women being less likely confident. Participants with a diagnosis of psychosis and attending normal CMHTs were more likely to indicate a desire to increase computer use than both those without psychosis and those with psychosis in the EI team. BME significantly predicted location of computer use, with non-white participants more likely to report using computers outside of their own homes.

**Table II:** Logistic regression results.

	Mobile phone	Computer	iPhone	BlackBerry
Familiarity	–	–	AGE: wald= 5.59*	AGE: wald = 5.18*
Access	–	AGE: wald = 4.52*	AGE: wald = 4.76*	AGE: wald = 9.38**
Confidence	GENDER: wald = 3.81*	AGE: wald = 9.95**	–	–
Desire to increase use	–	PSYCHOSIS GROUPING: wald = 3.90*	–	–
Frequency	AGE: wald = 7.05**	–	–	–
Location	–	BME: wald = 8.64**	–	–

Note: The significant predictor variables are capitalised.

* *p* < 0.05.

** *p* < 0.01.

## Discussion

Technology access and use in this sample were very similar to that of the general population: mobile phone access and confidence is 91% amongst the general population, and 78% of households in the UK own a PC or laptop (OFCOM, 2011). Additionally 27% of people in the UK now own a smartphone (OFCOM, 2011). These results are in contrast to the finding that those with medical conditions engaged with technology less than others (Wang et al., 2011). This might be the case because physical rather than mental conditions are more of a barrier in terms of accessing technology. Furthermore, this highlights that physical conditions are not always comparable to mental health problems. However, other previous findings which show that older and minority populations tend to have poorer access to technology (Fox & Vitak, 2008; Wang et al., 2011) are repeated amongst the sample studied here.

### Predictors of technology use and access

Those with higher age had lower knowledge, access and use for a number of items, which is in agreement with much of the previous literature – for example, Internet access, mobile phone use and smartphone use in the UK is considerably lower amongst older individuals (OFCOM, 2011; Office for National Statistics, 2011). Older people amongst the general population are also far less likely to report being confident when using the Internet (OFCOM, 2011), a finding which is reflected in our own sample. Women tended to be less confident than men when using mobile phones; again this is not surprising given that gender differences in confidence are well established with regard to technology use (Comber et al., 1997; Li & Kirkup, 2007). We found that BME groups reported using computers somewhere other than their own home more frequently than white participants. This is another result which is not unexpected, as black people in the USA are known to have lower rates of computer ownership than their white counterparts (US Bureau of Labour Statistics, 2007). This is a particularly important finding because any new introduction of HIT in mental health is likely to involve the exchange of very personal and private information. Consequently introduction of new HIT in mental health should consider how black minority and ethnic groups may need additional support relating to the provision of technology so that this group are not excluded.

### Can't surf or won't surf?

Despite the broad similarities between our survey and that in the general population there were some important differences. For instance a recent OFCOM (2009) survey found that self-exclusion was the main reason for non-use of the Internet amongst the general population, and that self-excluders tended to be older. This trend was not repeated amongst our mental health sample: participants experiencing psychosis who were attending standard CMHTs, and were therefore older than those in the EI group, reported a significant desire to increase their use of computers. It is interesting to note that participants in this group did not express a desire to increase their use of other technologies. As the question was hypothetical, i.e. assuming the skills and hardware were available, we might conclude that service users are not as interested in engaging with other technologies such as smartphones. From this finding we can draw two conclusions: (1) The preferred medium for engaging with HIT in this group is via computers and (2) That cost or lack of skills – not indifference – are the reasons why this group are not engaging with computers. It is interesting to note that this was only true for the older psychosis group. This demonstrates that the result is not solely related to age, as those of similar age but different diagnosis did not indicate a desire to increase computer use. One might explain this as an issue of education and employment – the onset of psychosis frequently disrupts education and employment rates of those with psychosis are low (Rinaldi et al., 2010). Consequently many of those in the older psychosis group might have little experience in using computers as compared to those in the non-psychosis group.

## Limitations

The study aimed to test those who have a longer term mental health problem and higher levels of disability (those with psychosis) who mainly use community mental health services (EI and CMHTs) as they are likely to be most in need of continued use of electronic personal health records. We can therefore not draw conclusions on those with less severe illnesses or those who are likely to use services more intermittently. However, there was sufficient statistical power to draw conclusions regarding the variables of diagnosis and BME that were of most interest. We were also unable isolate the effects of advanced age (over 65 years) on technology use and access – future research might address this issue. Any research involving technology is hampered by the transience of many technological devices, and this study is no different – from the time of data collection to submission, sales of Tablets and Android phones have substantially increased. As a result it is recommended that studies continually assess access and use as new technologies emerge which may influence engagement with HIT, so that service users obtain the maximum benefit from these technologies.

### Clinical and service implications

Healthcare providers should address the technology gap between older and younger service users so that older individuals experiencing psychosis are able to benefit from HIT. We therefore recommend the following to achieving maximum benefit:

(1) *Computer-based HIT is the most appropriate format* because access and confidence with computers are high, and computers represent the most versatile and private method of engaging with HIT.
(2) Service users must be provided with the equipment and skills required to engage with such HIT.
(3) Older service users require more support than younger service users.
(4) Particular attention should be given to BME groups when providing technological equipment.

Strategies for HIT introduction should take heed of the above if the large investments by both UK and US governments are to pay dividends. The benefits are potentially far-reaching, but it must be remembered that technological advances in themselves will not necessarily lead to the improvements suggested by policy makers: an appreciation of consumer appetites, access and skills regarding new technology is vital. This will ensure that resources are appropriately allocated to those who are the most “technologically poor”.
